# Supplementary figures and images for: RIP3 and MLKL regulate Hepatic ER stress in alcohol-associated liver disease and pharmacological ER stress models: insights beyond necroptosis
Source: bioRxiv. 2025 Sep 25:2025.09.24.677754. Preprint. [Version 1] doi: 10.1101/2025.09.24.677754 (PMC12485782; doi:10.1101/2025.09.24.677754)

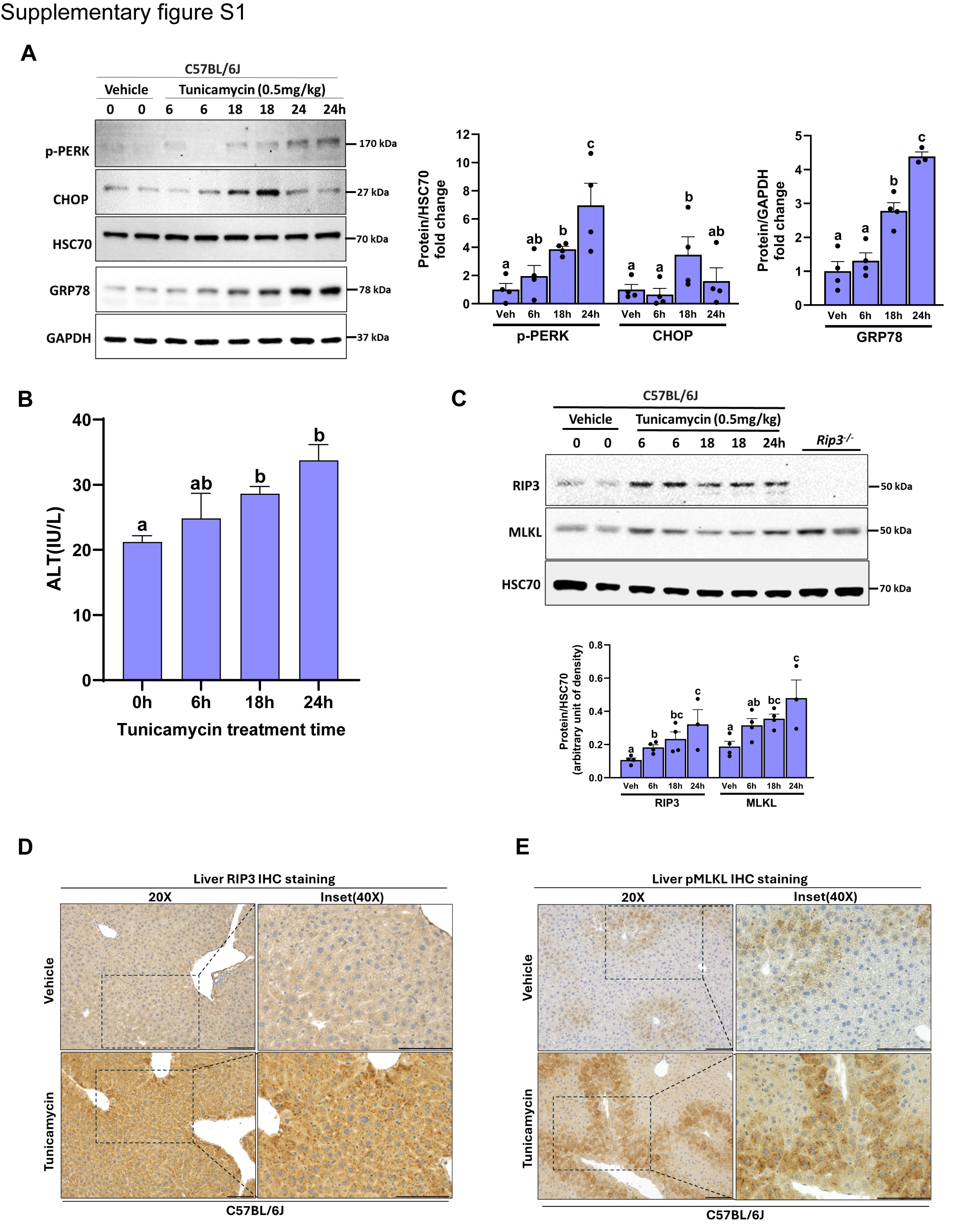

Supplement: Supplement 1 [file media-1.jpg]

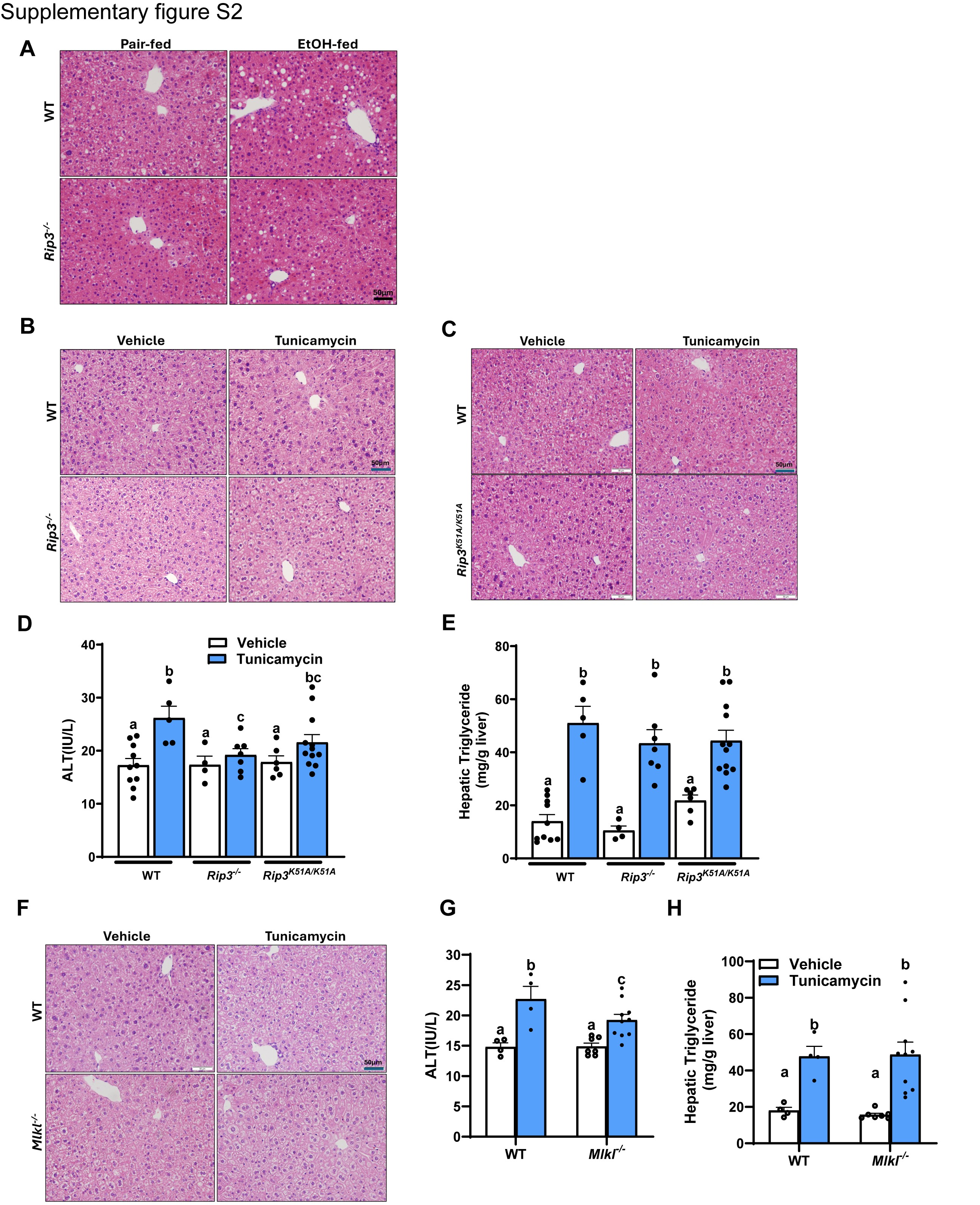

Supplement: Supplement 2 [file media-2.jpg]
